# Incidence of vertebral fractures in calcium and vitamin D-supplemented postmenopausal Brazilian women with osteopenia or osteoporosis: data from Arzoxifene Generations Trial

**DOI:** 10.1590/2359-3997000000141

**Published:** 2016-01-01

**Authors:** Henrique Pierotti Arantes, Suely Godoy Agostinho Gimeno, Alan Y. Chiang, John P. Bilezikian, Marise Lazaretti-Castro

**Affiliations:** 1 Unidade de Metabolismo Ósseo e Mineral Departamento de Endocrinologia Universidade Federal de São Paulo São Paulo SP Brasil Unidade de Metabolismo Ósseo e Mineral, Departamento de Endocrinologia, Universidade Federal de São Paulo (Unifesp), São Paulo, SP, Brasil; 2 Departamento de Medicina Preventiva Unifesp São Paulo SP Brasil Departamento de Medicina Preventiva, Unifesp, São Paulo, SP, Brasil; 3 Eli Lilly Company Indianapolis Indiana United States Eli Lilly Company, Indianapolis, Indiana, United States; 4 Department of Medicine College of Physicians and Surgeons Columbia University New York NY United States Metabolic Bone Diseases Unit, Division of Endocrinology, Department of Medicine, College of Physicians and Surgeons, Columbia University, New York, NY, United States

**Keywords:** Aging, Brazil, prospective study, risk factors, vertebral fractures

## Abstract

**Objective:**

Vertebral fracture is the most common osteoporotic fracture, affecting quality of life and increasing mortality. Epidemiological data on incidence of vertebral fracture are scarce in Brazil and throughout Latin America. Our aim was to determine vertebral fracture incidence and risk factors in a female Brazilian population.

**Subjects and methods:**

Postmenopausal women with low bone mass were studied from the Brazilian placebo group of Arzoxifene Generations Trial (n = 974), followed for up to 5 years. The primary endpoint was new vertebral fractures, detected by X-Ray. Experimental design defined two strata: A. Osteoporosis or previous vertebral fracture with osteopenia; B. Osteopenia without previous fracture. Previous fracture, T-score, ionized calcium, alkaline phosphatase, creatinine and glucose were analyzed at baseline. Crude and adjusted incidence rates of vertebral fractures were estimated and Poisson regression model was used.

**Results:**

Incidence rate was 7.7 (95% CI of 5.4 to 10.9) per 1,000 person-years (PY), increasing as a function of age. Women with new vertebral fractures had higher prevalence of previous nonvertebral fracture after menopause, were older and had lower lumbar spine (LS) T-score. Fracture risk increased by 46% for each unit reduction in LS T-score. Variables correlated with new vertebral fracture were age (p = 0.034), LS T-score, stratum A (p = 0.001 for both) and previous nonvertebral fracture after menopause (p = 0.019). In the final model, LS T-score was the strongest predictor.

**Conclusions:**

Incidence rate of vertebral fracture of 7.7 per 1,000 PY. Age and previous fractures were associated with new vertebral fracture, but LS T-score was the most important predictor.

## INTRODUCTION

Osteoporosis is characterized by low bone mass and microarchitectural abnormalities, that together increase fragility fracture risk (1). It is a serious public health problem that will get worse with the gradual aging of the world’s population (2). Vertebral fractures, a hallmark of osteoporosis, are the most common type of osteoporotic fracture (3), although only 1/3 of them are detected by an overt clinical event such as acute back pain (4). Besides, many vertebral fractures seen by X-ray or other imaging modality are not reported. In Latin America, 46.5% of vertebral fractures identifiable by X-ray are not recognized (5). However, vertebral fractures, even if asymptomatic, are associated with an increase in morbidity and mortality (6-11).

Knowledge of the epidemiology of osteoporotic fractures is of fundamental importance for the planning of preventive actions and treatment strategies, but these data are scarce, not only in Brazil but in all of Latin America. Brazil has a complex ethnic background with high level of miscegenation, resulting in a demographic blending of Native American Indians, Africans and Latino-Europeans who immigrated in the last few centuries, with almost 24 million people over 60 years old (12). It is estimated that by 2060, the population over 60 will increase by more than 3-fold, to 73.5 million (12). Thus, acknowledged risk factors for osteoporotic fracture gleaned from more homogeneous populations may not apply to Brazil.

The placebo arm of *Arzoxifene Generations Trial *(13), which had fracture detection as a main outcome measure, provided a convenience sample to investigate the incidence of osteoporotic fractures in naïve Brazilian postmenopausal women living in different regions of the country. To our knowledge, this is the first study to investigate vertebral fracture incidence in almost 1,000 postmenopausal women with low bone mass, aged between 60 to 85 years old, from multiple centers in Brazil. The results highlight the importance of several risk factors in this cohort that was followed prospectively for 5-years.

## SUBJECTS AND METHODS

This protocol was approved by the Ethics Committee of São Paulo Federal University (CEP 0972/2015). The placebo data were obtained from the Brazilian arm of the *Arzoxifene Generations Trial *(n = 974), funded by Eli Lilly and Company; ClinicalTrials.gov number, NCT00088010, which comprised of postmenopausal women with low bone mass, 60-85 years old, followed for up to 5 years, from 6 Brazilian cities distributed in 3 Brazil regions: Northeast (Recife and Salvador, n = 161), Southeast (Rio de Janeiro and Sao Paulo, n = 723), and South (Curitiba and Porto Alegre, n = 90). In this trial, women were divided in two strata: Stratum A (n = 648) was comprised of women with a femoral neck or lumbar spine with osteoporosis (bone mineral density (BMD) T-score of -2.5 or less (73.4% of this stratum) or women with a vertebral fracture at baseline and osteopenia (26.6% of this stratum). Stratum B (n = 326) was comprised of women whose femoral neck and lumbar spine osteopenia (T-scores were between -1.0 and -2.5) without a vertebral fracture at baseline. New vertebral fractures were assessed by lateral spinal radiographs at 6, 12, 24, 36 and in the last visit (at 60 months) for those patients in Stratum A and at baseline, 36 months and in the last visit (at 60 months) for those in Stratum B. Follow-up of this protocol was designed until 60 months or until the first fracture event (vertebral or nonvertebral). For example, if a patient had a fracture after one year of follow-up, the time to fracture was used to evaluate the incidence of fracture. Spinal radiographs were assessed independently by two readers at a central facility (Synarc) using a semiquantitative scale (14). This method is more efficient to perform than other methods of vertebral fracture assessment, suited to both clinical therapeutic efficacy trials and epidemiological research studies. It is easy to implement in clinical practice, as well as demonstrating excellent inter-reader reliability, as shown in a recent Brazilian paper, with agreement of 95% between interreader (15). The evaluator scale is graded from normal (grade 0) to severe (grade 3). Grade 1 (mild) vertebral fracture corresponds to a 20-25% reduction in anterior, middle, and/or posterior height compared to normal adjacent vertebrae or compared to expected height of the vertebral body based on experience. Grade 2 (moderate) vertebral fracture is a 25-40% reduction in vertebral height, while grade 3 (severe) vertebral fracture is a more than 40% reduction in vertebral height.

A new vertebral fracture was defined as a change from normal to grade 1, 2 or 3 on any subsequent radiograph. If two readers did not agree, the final determination was adjudicated by a third reader. Other details of the design of the study, recruitment, as well as exclusion criterion were previously published (13). All patients received daily supplements containing approximately 500 mg of elemental calcium and 400 to 600 IU of vitamin D. The primary endpoint analyzed in this study was new morphometric vertebral fractures, as assessed by X-Ray.

Baseline clinical and laboratories parameters were analyzed, including age, postmenopausal duration, previous fracture in the last 5 years or fracture after menopause. Fasting measurements were made of the following indices: creatinine, glucose, ionized calcium, alkaline phosphatase, 25 hydroxyvitamin D (DiaSorin (formerly Incstar Corporation, Stillwater, MN)), and bone turnover markers (P1NP and CTX) in a subset of patients (n = 307). BMD at the proximal femur and lumbar spine was evaluated by dual-energy X-ray absorptiometry (DXA). We had no access to smoking and alcohol abuse prevalence in these patients.

Statistical analyses were performed using STATA software (version 10.0, Stata Corp. College Station, USA). Mean and Standard Deviation (SD) were calculated for each continuous variable and percentage for categorical ones. Chi-square or Fisher’s Exact Test for categorical variables and Student’s *t*-test for continuous variables was used to compare differences in the baseline measures from patients with new vertebral fractures to those who did not. Crude and adjusted incidence rates of vertebral fractures were estimated by point and 95% confidence interval according to age and lumbar spine T-score categories. Incidence rate ratios were obtained to evaluate any association between risk factors and new vertebral fracture. The influence of the variables measured on the risk of incident fracture was analyzed using a Poisson regression model and the best fitting model obtained. All variables with a p-value of 0.20 in the univariate analysis were included in the regression full model. Only the statistically significant variables remained in the final model. A p-value of < 0.05 was considered to be significant.

## RESULTS

During a follow-up period of up to 5 years, 31 new vertebral fractures were detected. Table 1 shows the baseline characteristics of patients with and without new vertebral fractures.

**Table 1:** Baseline characteristics of postmenopausal women by new vertebral fracture

Variable	Overall	N	With new vertebral fracture	N	Without new vertebral fracture	N	p-value
**Age, years (SD)**	**67.1 (5.2)**	**974**	**69.0 (5.1)**	**31**	**67.0 (5.2)**	**943**	**0.034**
BMI, kg/m^2^	26.9 (4.4)	974	26.6 (4.0)	31	26.9 (4.4)	943	0.670
Previous vertebral fracture, n (%)	(14%)	974	(26%)	31	(14%)	943	0.062
Previous nonvertebral fracture, n (%)	(30%)	974	(45%)	31	(30%)	943	0.065
**Previous nonvertebral postmenop. fracture n(%)**	**(4%)**	**974**	**(13%)**	**31**	**(4%)**	**943**	**0.019**
Previous fragility fracture in the last 5 y	(16%)	974	(23%)	31	(16%)	943	0.172
**T-score spine**	**-2.5 (1.0)**	**974**	**-3.1 (0.8)**	**31**	**-2.5 (1.0)**	**943**	**0.001**
T-score neck	-1.8 (0.7)	974	-1.8 (0.7)	31	-1.8 (0.7)	943	0.705
T-score total hip	-1.4 (0.9)	869	-1.6 (0.8)	30	-1.4 (0.9)	839	0.243
Calcium, mmol/L	2.42 (0.09)	974	2.40 (0.08)	31	2.42 (0.09)	943	0.160
Glucose, nmol/L	5.6 (1.71)	937	5.2 (0.9)	30	5.6 (1.7)	907	0.218
Alkaline phosphatase, U/L	83.9 (23.0)	974	80.4 (25.6)	31	84.0 (22.9)	943	0.384
25OHD, nmol/L	68.02 (22.16)	969	72.7 (21.4)	30	67.8 (22.2)	939	0.229
P1NP, μg/L	50.1 (20.6)	307	56.1 (26.0)	14	49.8 (20.3)	293	0.270
CTX, ng/mL	0.606 (0.270)	307	0.672 (0.282)	14	0.602 (0.269)	293	0.349
Creatinine, μmol/L	69.5 (13.5)	974	69.1 (12.2)	31	69.5 (13.5)	943	0.893

**Figure 1:**
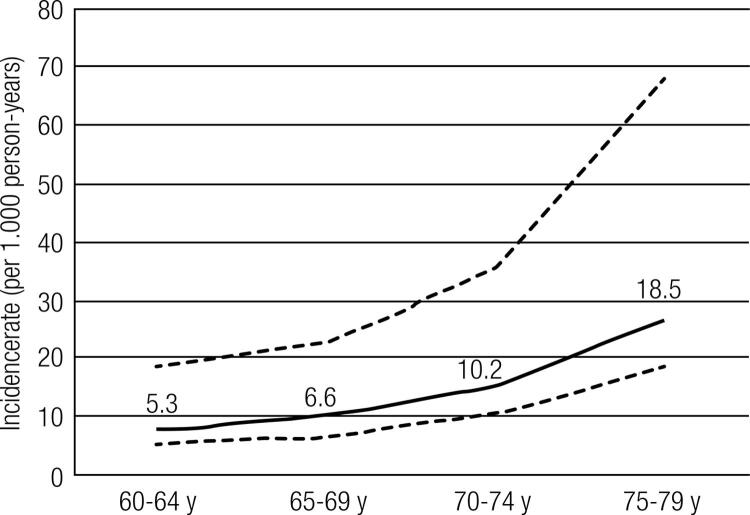
Incidence trends of vertebral fracture (by 1,000 person-years) according to different age groups. Dotted line represents 95% CI. Patients aged between 80-85 years were excluded from this analysis due to lower number of patients (n = 21).

Postmenopausal women with incident vertebral fractures were older, had lower lumbar spine T-scores and had a prevalence of previous nonvertebral fracture that was three times greater than the prevalence of previous nonvertebral fracture in patients without incident vertebral fracture. The distribution of patients with new symptomatic or non-symptomatic vertebral fracture was not associated with the grade of the fracture. The overall incidence rate of vertebral fractures, as determined by the semiquantitative scale of Genant and cols. was 7.7 (95% CI of 5.4 to 10.9) per 1,000 person- years (PY), increasing as a function of age. Patients between 75-79 years had a 3.5 fold higher incidence rate ratio of vertebral fracture as compared to the younger group 60-64 years (p = 0.02), as shown in Figure 1. We could not ascertain fracture incidence accurately in those between 80 to 85 years because there were only 21 subjects in this age bracket.

**Figure 2:**
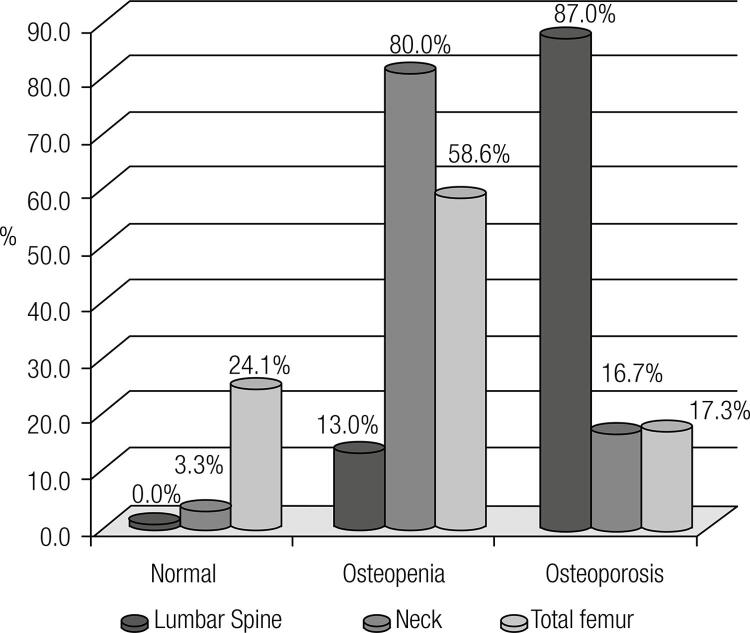
Percentage of patients with new vertebral fracture (n = 31), according to WHO T-score classification of BMD at different sites.

As expected, there was an inverse correlation between lumbar spine T-score and the risk of incident vertebral fracture. For each SD decrease in lumbar spine T-score, the risk of fracture increased by 46% (CI 22 – 62%; p = 0.001). Moreover, women with osteoporosis or low bone mass with previous vertebral fracture (Stratum A) had 3.9 times more new fractures than those with osteopenia (Stratum B) (p = 0.001). Twenty-six point six percent of all sustained vertebral fractures occurred in patients without a densitometric osteoporosis (Stratum A). Nevertheless, of all new vertebral fractures, 87% occurred in patients with osteoporosis (Figure 2).

Based on the Poisson regression analysis, the variables correlated with new vertebral fracture included age (p = 0.034), lumbar spine T-score (p = 0.001), stratum A (p = 0.001) and previous nonvertebral fracture after menopause (p = 0.019). For the regression full model, apart from the above-mentioned variables, the following data, although not statistically significant were, nevertheless, of interest: a history of fragility fracture in the last 5 years (p = 0.172), previous vertebral fracture (p = 0.062), previous nonvertebral fracture (p = 0.065) and serum calcium concentration (p = 0.160). In the final model, the variable that contributed most to the prediction of new vertebral fracture was the lumbar spine T-score (incidence rate ratio of 1.46, 95% CI 1.22 to 1.62, p-value of 0.001).

## DISCUSSION

It is important to emphasize that this study is not strictly an epidemiologic one. These data came from a placebo group of a randomized clinical trial, which has specific inclusion and exclusion criteria that may not be reflected in the general population. As such, we cannot rule out an element of selection bias. Other limitations refer to the fact that only the first new fracture during the study was taken into account. Besides, all participants received calcium and vitamin D during the protocol. On the other hand, an observational study in a population with known high fracture risk would be very difficult be obtain approval for with calcium and vitamin D only.

To our knowledge, this is the first Latin America study to evaluate osteoporotic vertebral fracture incidence assembled from centers distributed throughout the same country. The estimated incidence rate was 7.7/1,000 PY. The incidence of vertebral fractures in postmenopausal women with low bone mass increased with age, being 3.5 fold higher in patients aged between 75 to 79 (incidence rate of 18.5/1,000 PY), as compared to the group, 60 to 64 years old (incidence rate of 5.3/1,000 PY). This increase in fracture risk is similar to The European Prospective Osteoporosis Study (EPOS) (3), which showed an increase of 3.1 fold in the group aged between 75 to 79 compared to 60 to 64 years. In addition, the results are concordant with the Rotterdam study (16), which found that patients aged 75 years or more had 2.5 fold higher vertebral fracture incidence than the group aged between 55 to 65 years old. These data demonstrate the importance of age as a key determinant of fracture risk.

Based on population projection from the Brazilian Institute of Geographic and Statistics (IBGE in Portuguese) (12), it is expected that the female population between 60 to 79 years old will triple from 2013 to 2060. Assuming that in Brazil 2/3 of this population has low bone mass or osteoporosis (17) and based on our data, a reasonable estimate projects an increase in the number of new vertebral fractures from 60,000 in 2013 to more than 188,000 in 2060.

The estimated incidence rate of 7.7/1,000 PY seems to be somewhat lower than the EPOS study (3) (10.7/1,000 PY) and the Rotterdam study (16) (14.7/1,000 PY), especially if one considers that our population was pre-selected and had low bone mass. Nevertheless, these studies utilized a more stringent criterion for vertebral fracture (reduction of 20% and 15% of vertebral height, respectively). In the EPOS Study, using a more stringent criterion of 25% reduction in vertebra height, the incidence rate falls to 8.7/1,000 PY. Both Rotterdam and EPOS studies used a morphometric method of McCloskey-Kanis and, thus, could account for these different results. In our study, the Genant’s semiquantitative method was used to define new vertebral fracture. Notwithstanding a high concordance between these methods in cases of moderate and severe fractures, morphometry is associated with a more false positive diagnosis of mild fractures than the semiquantitative approach (18,19).

Ross and cols. studied 887 Hawaiian women of Japanese ancestry, between 43 and 80 years of age, followed for up to 4.7 years (20). They found an overall incidence of 15.0/1,000 PY. Nevertheless, a reduction of 15% in vertebral height was considered as fracture, which is less stringent than the criterion used here of 20%. It is important to point out, however, that our protocol was designed to include the first event only, refracturing not being taken into account. In the Rotterdam Study, one-fourth of all new fractures were due to refracture (16).

Two hundred thirty two sites from 23 different countries were involved in the *Arzoxifene Generations Trial* (13). In the placebo group, the incidence of new vertebral fractures was 4.3% in all countries, confirming that it was slightly higher when compared with 3.2% found in the Brazilian arm. The mean age was similar in all global regions (Europe, North America, South America and other), as well as in Brazil. This interesting comparison takes into account similar inclusion and exclusion criteria, X-rays analysis to define new vertebral fracture as well as the same window of time.

The overall incidence of clinical (symptomatic) vertebral fracture in a population-based epidemiologic study in Rochester was 1.45 per 1,000 person-years (21). It is well established that the majority of vertebral fracture is asymptomatic, and literature considers that only one-third of them is symptomatic (4). Nevertheless, we found an even lower rate of this kind of fracture, with only one-fifth of cases symptomatic in our set. Considering this, the incidence rate of clinical vertebral fracture in our study is very similar to the Rochester experience (21).

Only two studies are available on vertebral fracture incidence in Brazilian cohorts (22,23) with different designs, and they cannot be properly compared with each other or with our study. One of these studies was carried out in an Osteoporosis Outpatient Clinic from a School-Hospital in Sao Paulo, and found an incidence rate of 41.7 fractures per 1,000 person-years, 70% of which were vertebral fractures (22). This rate was determined from 275 postmenopausal women evaluated at baseline and 5 years later. In that study, patients with fracture were older than our patients. They were at greater fracture risk also because of a referral bias (a tertiary care center), a higher prevalence of previous fracture. However, risk factors for fractures were very similar to ours.

Another Brazilian population-based study published recently (23) found a vertebral fracture incidence of 40.3/1,000 person-years fracture in a population > 65 years living in a district of Sao Paulo, which is more than five times higher incidence than we found. In this study, women were older than ours (72.9 *vs* 67.1 years old, respectively), with 70% older than 70, whereas only 30% of our patients were in this older age range. Femoral neck BMD was substantially lower in that cohort than in ours (0.656 *vs* 0.714 g/cm^2^, respectively). Other differences included a high refracture rate of 43.2% in their patients. On the other hand, both studies found similar risk factors for vertebral fracture in postmenopausal Brazilian population (age, lumbar spine BMD and previous fracture). Another point that is noteworthy is that during the *Arzoxifene Generations Trial*, all postmenopausal women received supplementation with calcium (500 mg daily) and vitamin D (400 to 600 IU), which may have reduced incidence of fractures. Notwithstanding, a meta-analysis showed that calcium and vitamin D supplementation was ineffective in preventing vertebral fractures (24).

Apart of age and previous fracture, the lumbar spine T-score was the most relevant risk factor for a new vertebral fracture. This strong association between lumbar spine T-score and vertebral fracture may be due to the rapid loss of trabecular bone in the first years after menopause, being revealed most clearly at a trabecular site (i.e. lumbar spine). Other point that may help to account for this strong association is that our relatively young population (< 70 years) is less likely to have osteoarthritis, which can artifactually overestimate spine BMD. Interestingly, we did not find any association between femoral neck or total hip BMD with vertebral fracture (Figure 2). Local femoral cortical bone thickness and density declines later than trabecular bone of the lumbar spine. In this regard, it is not surprising that femoral BMD is less predictor of vertebral fracture in younger patients. This question could be evaluated in future studies, using FRAX with and without femoral neck BMD input.

An interesting finding to be underscored is the observation that stratum A had 90.3% (n = 28 in 31) of new fractures, illustrating not only the importance of osteoporosis but also of osteopenia with previous fracture as risk factors for a new vertebral fracture.

One of the strengths of this study is that it encompasses many different regions in Brazil over a reasonably long period of time. It constitutes the first study of its kind in Latin America. Another strength is that vertebral fractures were analyzed at a specialized, independent, central facility.

In conclusion, in this selected Brazilian postmenopausal population with osteopenia or osteoporosis, the incidence rate of vertebral fracture was 7.7 per 1,000 person-years, increasing from 5.3 in patients aged between 60 to 64 years old to 18.5 in older than 75 to 79 years old. Age and previous fractures were associated with new vertebral fracture, but lumbar spine BMD was the most important predictor. For each SD decreased at BMD in lumbar spine T-score, there was an almost 50% increase in the risk of fracture.
